# Retroperitoneal Spindle Cell Tumor: A Case Report

**DOI:** 10.3389/fsurg.2021.764901

**Published:** 2021-12-15

**Authors:** Hao Hua, Zhiwei He, Linhan Lei, Huahua Xie, Zilei Deng, Zili Cheng, Shi Zuo, Chengyi Sun, Chao Yu

**Affiliations:** Department of Hepatic-Biliary-Pancreatic Surgery, The Affiliate Hospital of Guizhou Medical University, Guiyang, China

**Keywords:** retroperitoneal, spindle cell tumor, myofibroblastoma, surgery, case report

## Abstract

Spindle cell tumor is very rare. Herein, we report a case of retroperitoneal spindle cell tumor in a 52-year-old female. The patient first presented with a complaint of persistent pain in the right upper abdomen. In the follow-up, a CT scan was performed and showed a retroperitoneal soft tissue density mass measuring 11 cm in diameter. Then, a subsequent operation was performed, and we completely removed the tumor and partially invaded lesions. The tumor was histologically diagnosed as a spindle cell tumor. Therefore, it is imperative for us to enhance the understanding of this seldom found tumor. Surgery remains the best option for treatment.

## Introduction

Various types of human tumors can present as spindle cells, such as poorly differentiated epithelial-derived tumors, gastrointestinal stromal tumors, mesenchymal tumors, and neurogenic tumors (1, 2). Spindle cell tumor, characterized histologically by a mixture of fat cells and fibroblast-like spindle cells in a matrix of collagen and mucoid material, is rare, and its incidence is low and can occur in human soft tissue, bone, or in any part of the human body, such as the retroperitoneal space. Its morphological appearance can be carcinomatous or neoplastic. Because of its rarity, there have been no adequate studies on its clinicopathological characteristics and diagnosis scheme. Herein, we are present a case of retroperitoneal spindle cell tumor to raise awareness on this seldom found tumor.

## Case Presentation

**Figure 1:**
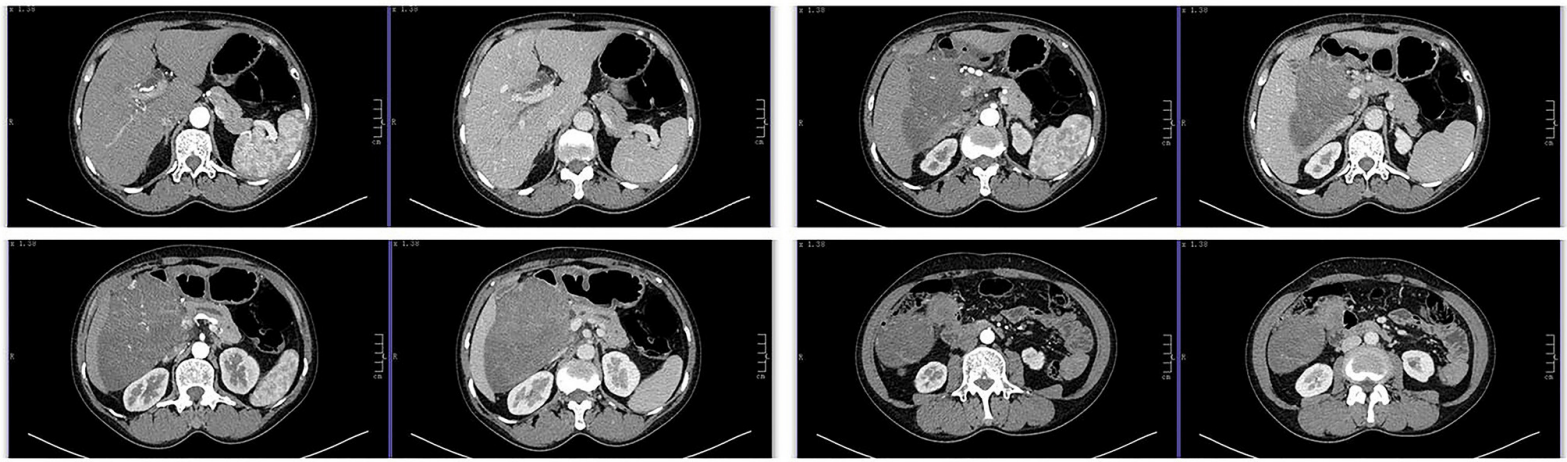
Computed tomography (CT) scan showed a retroperitoneal soft tissue density mass, 11 cm in size, which is closely related to the liver, first and third hepatic portals, portal vein, hepatic artery, and pancreas.

A 52-year-old female patient presented with a complaint of persistent pain of 1 month duration in the right upper abdomen. There was no history of previous abdominal trauma, bleeding, or family history of cancer. On clinical examination, there was slight tenderness in the right upper abdomen. Serum α-fetoprotein, carcinoembryonic antigen, carbohydrate antigen 125, and carbohydrate antigen 19-9 were normal. There was no obvious clinical history of malignancy. Enhanced upper abdominal computerized tomography (CT) showed a soft tissue density mass 11 cm in diameter (Figure 1). Therefore, we speculated that the retroperitoneal tumor was a benign tumor. Additionally, pathological results of pre-operative puncture were inflammatory myofibroblastoma and nodular fasciitis. Then, we chose to perform surgery.

**Figure 2:**
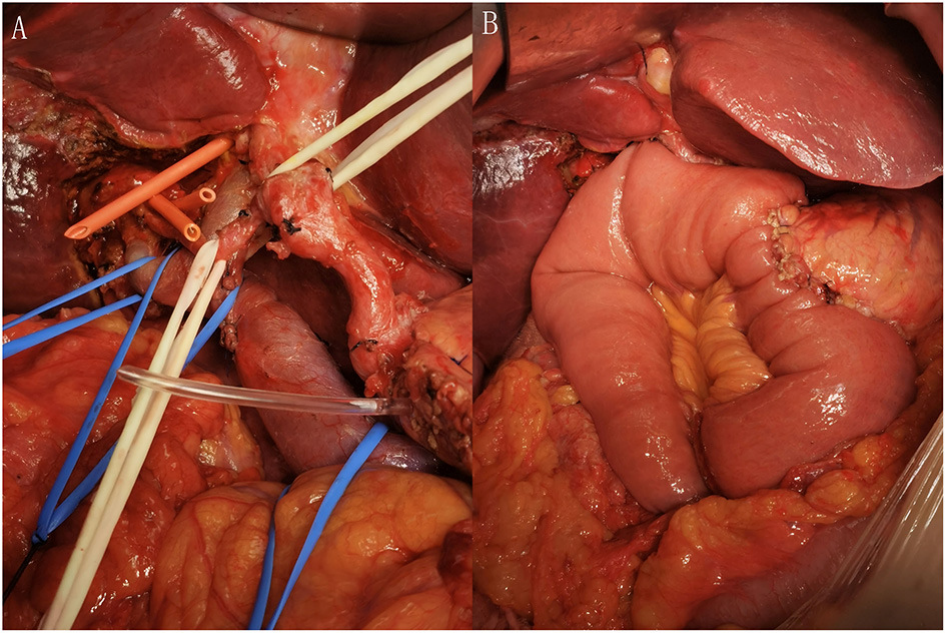
**(A)** After removal of the tumor from the abdominal cavity and **(B)** after the reconstruction of digestive tract.

The findings of intraoperative exploration revealed that the tumor was involved in the right hepatic artery, main portal vein, right portal vein, gallbladder, gastric antrum, duodenum, and head of pancreas. According to the results, we performed pancreaticoduodenectomy, perihilar hepatectomy, neoplasty of high bile duct, partial resection and repair of the main portal vein, partial resection and repair of the right portal vein, repair of the right hepatic artery, and reconstruction of the digestive tract (Figure 2). Finally, the operation was successful, lasting 7 h, and the intraoperative bleeding was 350 ml. The patient recovered well after the surgery.

**Figure 3:**
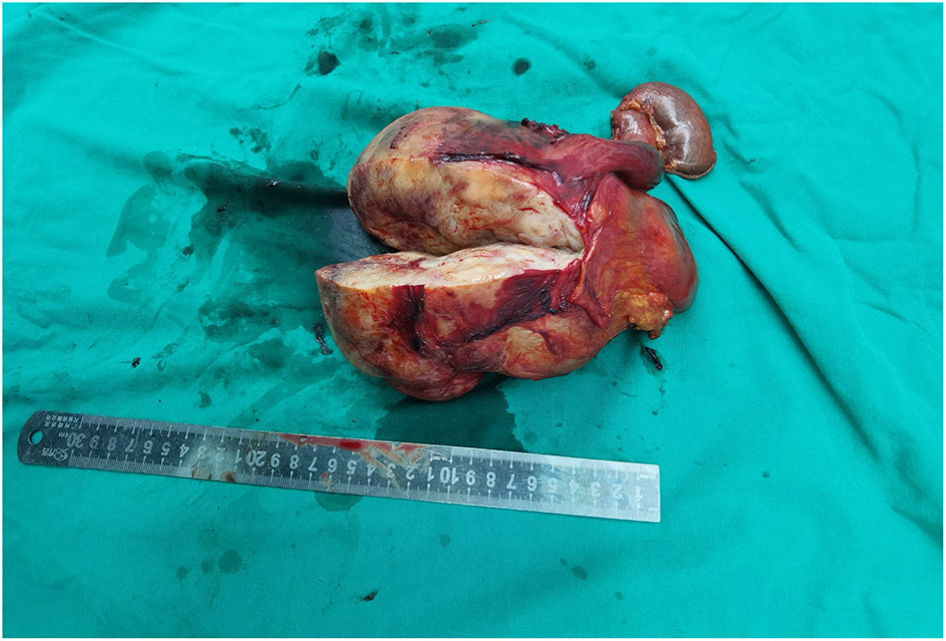
The surgical specimen showed that the tumor was a well-delineated solitary mass, and that the actual size was ~17 × 12 cm. The section of the tumor showed a pale and solid mass, which is relatively tough.

**Figure 4:**
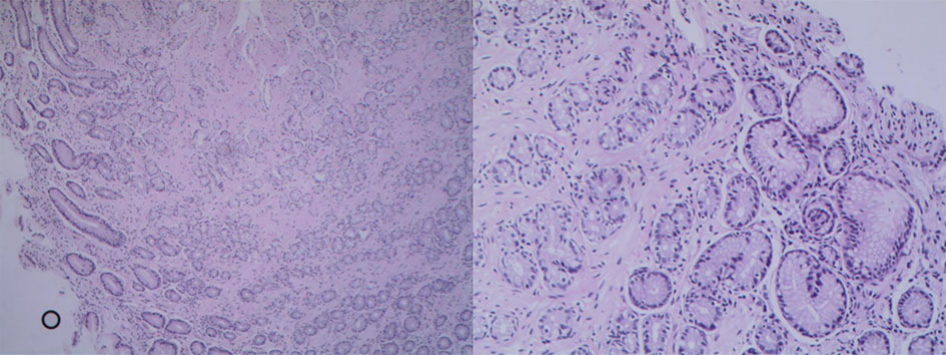
Pathological images showed spindle cells with involvement of the pancreas, duodenal serosa, and serosa of the gallbladder. No lymph node metastasis was found.

The tumor was solitary and solid with an off-white cross-section (Figure 3). The actual size of the tumor was ~17 × 12 cm. Microscopically, the tumor was predominantly made of spindle cells, and the margin of resection was negative (Figure 4). The tumor was diagnosed as a spindle cell tumor depended on these consequences.

## Discussion

To the best of our knowledge, soft tissue tumors usually occur in the limbs. Therefore, abdominal soft tissue tumors, especially retroperitoneal spindle cell tumors, are uncommon (3). Recently, there are still insufficient clinical research studies on spindle cell tumors. Spindle cell tumors, first reported by Weiss and Enzinger in 1896, originate from a variety of types of tumors, such as fibrosarcoma, gastrointestinal stromal tumor, and intra-abdominal desmoid tumor (4). Most of them occur at the young age of 20–40 years, occasionally appear in child, and their incidence has no significant differences between males and females (5, 6). In 1994, the WHO officially classified spindle cell tumors as soft tissue neoplasms.

Due to the diversity of pathological morphology, it was once named after inflammatory pseudotumor. A previous article has reported that the biological behavior of the tumor cells is inert, and is associated with distant metastasis, and that their prognosis is well (2). Clinical examination, imaging, and histology are non-specific in the evaluation of the uncommon tumor and distinguish it from other solid masses. Thus, pathological and immunohistochemical tests are critical to the definitive diagnosis of this kind of tumor. β-Catenin has an imperative role in the development of the differential diagnosis of spindle cell neoplasms, particularly in the abdomen (7–10). In a prior study, Torres reported that β-catenin nuclear staining is probably the single most vital feature and is fundamental for the diagnosis (11). Meanwhile, the study by Carlson indicated that nuclear immunoreactivity for β-catenin is supportive for the diagnosis of this kind of lesion (12). Also, immunohistochemical staining of CD34 and CD117 antibodies can be performed for differential diagnosis (13). Another research found that the application of Ki-67 for the identification of tumor cells with spindle cell morphologic characteristics of smooth muscle cells and fibroblasts is of great importance (14). In our case, the diagnosis still requires pathological and immunohistochemical confirmation. This is a unique case report not only because retroperitoneal spindle cell tumors are extremely rare, but also because the tumor was diagnosed as fibromatosis. However, identification of additional cases and further research studies on the diagnosis of spindle cell tumor are necessary.

The management of retroperitoneal spindle cell tumors is complicated and based on their clinical biological behavior. In this case, CT is effective in detecting the soft tissue density mass located in enterocoelia, which was suspected of local lesion. The pathological results of pre-operative puncture are different from the post-operative results mainly not only because of its rarity but also plenty of neoplasms have overlapping cytomorphologic features. Complete surgery is the main treatment for intra-abdominal spindle cell tumors. If the tumor invades other organs in the abdominal cavity, extensive radical resection may be carried out (15). In our case, the patient presented with a tumor with a maximum cross section diameter of 11 cm, but we successfully removed the tumor and reconstructed the digestive tract. Recurrence rates as high as 20–68% have been reported even after negative-margin excision of desmoid tumors, and the prognostic significance of microscopic positive margin remains unknown (16). Through following the treatment model of surgically removing the entire tumor with an adequate tumor-free margin, we thoroughly detected the tumor and part of the invading lesions, and to date the patient has been free of recurrence since the surgery.

In conclusion, due to being a rare disease, much more studies on retroperitoneal spindle cell tumors are needed to enhance the management of this lesion. Complete resection is the gold standard for this kind of rare disease.

## Data Availability Statement

The raw data supporting the conclusions of this article will be made available by the authors, without undue reservation.

## Ethics Statement

Written informed consent was obtained from the individual(s) for the publication of any potentially identifiable images or data included in this article.

## Author Contributions

HH prepared and wrote this article. ZD, HX, and LL were involved in managing the patient. ZC prepared the intraoperative pictures. CY revised the manuscript as well as acted as the corresponding author. ZH wrote and reviewed this manuscript. SZ and CY were the main surgeon. CS was involved directly in managing the patient. All authors contributed to the article and approved the submitted version.

## Funding

This research was financially supported by the funds from Science and Technology Planning Project of Guizhou Province, Science and Technology Cooperation Support of Guizhou Province [2021] General 080, Clinical Medical Research Center of Hepatobiliary Surgery of Guizhou Province, Science and Platform Talent of Guizhou Province [2017], Science and Technology Foundation of Health and Family Planning Commission of Guizhou Province [grant number: gzwkj2021-167], and Science and Technology Planning Project of Guizhou Province construction contract [2019].

## Conflict of Interest

The authors declare that the research was conducted in the absence of any commercial or financial relationships that could be construed as a potential conflict of interest.

## Publisher's Note

All claims expressed in this article are solely those of the authors and do not necessarily represent those of their affiliated organizations, or those of the publisher, the editors and the reviewers. Any product that may be evaluated in this article, or claim that may be made by its manufacturer, is not guaranteed or endorsed by the publisher.
